# A proposed case-control framework to probabilistically classify individual deaths as expected or excess during extreme hot weather events

**DOI:** 10.1186/s12940-016-0195-z

**Published:** 2016-11-15

**Authors:** Sarah B. Henderson, Jillian S. Gauld, Stephen A. Rauch, Kathleen E. McLean, Nikolas Krstic, David M. Hondula, Tom Kosatsky

**Affiliations:** 1 Environmental Health Services, British Columbia Centre for Disease Control, 655 West 12th Avenue, Vancouver, BC V5Z 4R4 Canada; 2 School of Population and Public Health, The University of British Columbia, 2206 East Mall, 3rd Floor, Vancouver, BC V6T 1Z3 Canada; 3 Center for Policy Informatics, School of Public Affairs, Arizona State University, Phoenix, AZ 85004 USA

**Keywords:** Extreme hot weather, Population mortality, Vulnerability, Case-control, Administrative data, Public health

## Abstract

**Background:**

Most excess deaths that occur during extreme hot weather events do not have natural heat recorded as an underlying or contributing cause. This study aims to identify the specific individuals who died because of hot weather using only secondary data. A novel approach was developed in which the expected number of deaths was repeatedly sampled from all deaths that occurred during a hot weather event, and compared with deaths during a control period. The deaths were compared with respect to five factors known to be associated with hot weather mortality. Individuals were ranked by their presence in significant models over 100 trials of 10,000 repetitions. Those with the highest rankings were identified as probable excess deaths. Sensitivity analyses were performed on a range of model combinations. These methods were applied to a 2009 hot weather event in greater Vancouver, Canada.

**Results:**

The excess deaths identified were sensitive to differences in model combinations, particularly between univariate and multivariate approaches. One multivariate and one univariate combination were chosen as the best models for further analyses. The individuals identified by multiple combinations suggest that marginalized populations in greater Vancouver are at higher risk of death during hot weather.

**Conclusions:**

This study proposes novel methods for classifying specific deaths as expected or excess during a hot weather event. Further work is needed to evaluate performance of the methods in simulation studies and against clinically identified cases. If confirmed, these methods could be applied to a wide range of populations and events of interest.

## Background

Extreme hot weather events have been associated with sharp increases in population mortality. The most dramatic examples include Chicago in 1995 [1, 2], Western Europe in 2003 [3–7], and Moscow in 2010 [8], but there are many examples of lesser impacts worldwide [9–14]. One hallmark of these events is that very few of the deaths meet the criteria [15] for being certified with ambient heat as the underlying cause [16–19], making it challenging to separate the excess heat-related deaths from the expected deaths that would have occurred regardless of temperature. A simple method for probabilistically identifying these excess deaths would be valuable for supporting epidemiologic analyses and improving public health outreach during future hot weather.

In the summer of 2009 the metropolitan area of greater Vancouver, Canada experienced an extreme hot weather event that resulted in a 40% increase in mortality over a 7-day period. However, only one of the deaths during this period included an International Classification of Diseases (ICD) code for ambient heat as the underlying cause. Rapid, case-only analyses conducted with the administrative vital statistics data indicated that mortality during the hot weather event was shifted towards deaths in the community, younger seniors (aged 65–74 years), densely populated areas, and more deprived areas [17]. Recent studies conducted in other urban areas have also indicated that a lack of residential greenness contributes to an increased risk of mortality during extreme hot weather [19, 20].

Here we leverage these known associations to probabilistically separate the excess deaths from the expected deaths using a novel resampling approach applied within a case-control framework. The reasoning is as follows: (1) some of the deaths that occur during an extreme hot weather event are expected, regardless of temperature; (2) those expected deaths are similar to deaths that occur during typical summer weather with respect to characteristics that describe the decedents, their residential neighborhoods, and the circumstances of their deaths; (3) if the expected deaths alone were to be included in a case-control analysis with typical weather deaths, the effects of variables known to be associated with heat-related mortality should be null; (4) if the expected number of deaths is repeatedly sampled from all deaths observed during the extreme hot weather event, deaths that are consistently present in non-null subsets are more likely to have occurred because of the heat.

## Methods

### Context

**Fig. 1:**
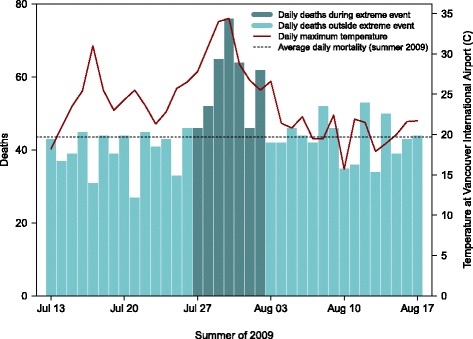
Daily time series of the greater Vancouver extreme hot weather event in the summer of 2009. The maximum temperatures measured at Vancouver International Airport are shown on the right-hand axis, and the 411 deaths included in the pool of cases are shown as the darker bars

The 2009 extreme hot weather event in greater Vancouver has been described in detail elsewhere [17, 20]. In brief, Vancouver is a coastal city with a population of 2.8 million residents and a mild climate. The average (standard deviation) high temperature on summer days (June through August) is 21 °C (3 °C) at Vancouver International Airport (YVR). The hottest 7-day period of 2009 occurred between July 27 and August 2, with high temperatures ranging from 26 °C to 34 °C and low temperatures ranging from 16 °C to 22 °C at YVR. Although these conditions may not seem extreme when compared with those experienced elsewhere, they were unprecedented in the history of the study area [20]. There were 411 adult deaths from all causes during this period, compared with an average (standard deviation) of 297 (22) during all other summer weeks of 2009–2012. Deaths started to increase above baseline on July 26, and the increase was sustained until August 2 with no clear evidence of mortality displacement (Fig. 1). Here we define the extreme event as July 27 through August 2 to be consistent with our previous case-only analyses, and we do not consider the impacts of air pollution because we have already reported that the ambient concentrations remained relatively low [17].

### Data sources

All summer deaths from 2009 to 2012 were extracted from the administrative vital statistics data received daily by the British Columbia Centre for Disease Control (BCCDC) to support its public health surveillance and protection mandates. The database contains fields for date of death, age, sex, location of death (including home, hospital, residential institution, or other), underlying cause of death (ICD, 10^th^ revision), and residential 6-digit postal code. The latter was used to geolocate every record and to assign variables for neighborhood population density, deprivation, and vegetative greenness. Population density estimates for the year 2010 were taken from the Gridded Population of the World (GWPv4) project, which models the global distribution of the human population at a spatial resolution of 30 arc seconds (~1km at the equator) using all available census data [21]. Deprivation quintiles were assigned from 2006 values of the Vancouver Deprivation Index (VANDIX), which was developed with local public health practitioners to reflect the variables most relevant to health in the city [22]. Neighborhood greenness was measured by the normalized difference vegetation index (NDVI), which was taken from the summer of 2009 Landsat 7 ETM+ composite for the region. The NDVI can take values from −1.0 to 1.0, with surfaces such as rock having values <0.1 and healthy forests having values >0.6 [23]. Previous work has shown that lower NDVI values are associated with increased land surface temperature and the urban heat island effect [24].

### Statistical approach

All 411 adult deaths that occurred from 27 July through 2 August 2009 were included in the pool of cases (Fig. 1). All 11,632 adult deaths that occurred during the summers (June, July and August) of 2010–2012 were included in the pool of controls. This control period was chosen (1) to ensure that all controls survived the 2009 event and (2) because there were no heat health emergencies in the greater Vancouver during those summers. The regional heat health emergency warning system was developed in response to the excess mortality observed during the 2009 event [20]. The case-control analyses were conducted using five variables that have previously been associated with mortality during extreme hot weather events: age; location of death; population density; neighborhood deprivation quintile (VANDIX); and neighborhood greenness (NDVI). The continuous age variable was categorized to <75 and ≥ 75 years because our previous work indicated that the lower age category was at higher risk during this event [17]. The NDVI values were normally distributed and used as a continuous variable in the analyses. The location of death was a factor variable with four categories, and death in hospital was used as the reference. Both VANDIX and population density were ordered quintiles that we used as continuous variables in the analyses. The population density was converted to quintiles because it followed no clear distribution and its peak was at the very end of its right tail, reflecting the dense urban population in some parts of the city.

The basic methodological framework was a logistic regression model into which 297 deaths from the pool of 411 cases and 1188 deaths from the pool of 11,632 controls were randomly selected, creating a 4:1 ratio of controls to cases. The expected number of 297 deaths was taken from the average mortality during typical summer weeks from 2009 to 2012. If the model was null, all of the cases were flagged as being selected into a null model; if the model was significant, all of the cases were flagged as being selected into a significant model. A model was defined as significant when any independent variable had a p-value less than the specified alpha value, as described in the following section on the univariate and multivariate modelling combinations. This process was repeated 10,000 times over 100 trials. We conducted 100 trials to evaluate the consistency of the results between trials.

Within each trial tallies were recorded reflecting (A) the number of times each case was randomly selected into a repetition, and (B) the number of times each case appeared in a significant model. After all repetitions were complete, the value for (B) was divided by the value for (A), resulting in a probability ratio of selection into significant models over all selections for each case. The cases were then ranked according to this value. The top 114 (411 total deaths – 297 expected deaths) were classified as the more probable excess deaths while the bottom 297 cases were classified as the more probable expected deaths. Once the 100 trials were complete, the percentage of trials in which a case was identified as a more probable excess death was calculated. The 114 cases that had the highest values were identified as the most probable excess deaths. The standard deviation of the ranking for each case across trials was recorded to evaluate the consistency of the results. We chose 10,000 repetitions to optimize the balance between the mean of these values and the computation time per trial.

### Modeling combinations

Within this framework there are modeling choices that can impact the final results. We used a series of 12 combinations to assess sensitivity to these choices, with variations in the (1) type of model, (2) evaluation of variable significance, (3) importance of coefficient direction, and (4) alpha value (Table 1). The model type was either univariate or multivariate. In the univariate combinations each of the five variables was evaluated separately over 10,000 repetitions for the selected cases and controls; then the significance and selection tallies were summed across variables before the probability ratio was calculated and the case ranking was performed. In the multivariate combinations all five variables were included in a single model over 10,000 repetitions. Within the multivariate models, the probability ratio was calculated using either (1) the tally of repetitions with at least one significant variable, or (2) the tally of the count of the number of significant variables in each model repetition. The importance of coefficient direction was also tested for both the univariate and multivariate combinations. The first variation counted any significant variable, regardless of its direction. The second variation only tallied significance if the coefficient was in the expected direction based on previous work, as follows: positive for the younger age category compared with the older age category; negative for increasing NDVI; positive for any location of death compared with death in hospital; positive for increasing deprivation; and positive for increasing population density. Finally, alpha values of 0.10 and 0.05 were tested for the significance threshold.

**Table 1:** Summary of variants for each combination and standard deviations (SD) for case ranks between trials

Combination	Significance tally	Coefficient in expected direction	Alpha	Mean of rank SD	SD of rank SD	Median of rank SD	Interquartile range of rank SD
Univariate Combinations							
#1	Summed across variables	T	0.10	36.94	12.09	40.37	17.50
** #2**	**Summed across variables**	**F**	**0.10**	**36.61**	**11.67**	**40.10**	**15.81**
#3	Summed across variables	T	0.05	41.12	12.68	44.44	17.72
#4	Summed across variables	F	0.05	41.05	12.71	44.40	18.18
Multivariate Combinations							
** #1**	**Count of significant variables**	**T**	**0.10**	**50.56**	**12.77**	**54.23**	**17.52**
#2	Count of significant variables	F	0.10	50.56	12.92	54.43	17.76
#3	Count of significant variables	T	0.05	60.07	14.76	64.25	18.56
#4	Count of significant variables	F	0.05	60.31	14.81	63.89	22.29
#5	At least one significant variable	T	0.10	89.74	13.62	93.17	15.21
#6	At least one significant variable	F	0.10	89.83	13.44	94.09	14.82
#7	At least one significant variable	T	0.05	66.83	18.16	74.09	27.79
#8	At least one significant variable	F	0.05	67.07	17.95	74.75	23.72

Bold highlighting indicates the univariate and multivariate combinations selected as the most promising combinations based on minimized variability in the ranking results

### Assessment of the results

Outcomes of the analyses were compared between the 12 combinations. The standard deviation of rank was calculated for each case across the 100 trials, and variability in the standard deviations for the 411 cases was used as an indicator of consistency across trials. The most consistent univariate and multivariate combinations were selected for more detailed description of the analytic results. We expected the probable excess deaths to be significantly different from the controls with respect to each of the five variables used in the analyses. Conversely, we expected the probable expected deaths not to be significantly different from the controls with respect to each variable. As such, statistical assessment of these assumptions was tested for each combination. A two sample *t*-test was used for the NDVI variable, chi-square tests were used for the categorical age and location of death variables, and the Wilcoxon Mann-Whitney test was used for the ordinal deprivation and population density variables. 7We also expected that the probable excess deaths would occur more frequently on the hotter days of the study period (Fig. 1), which we assessed for the most consistent combinations. Finally, the overlap in probable excess deaths was compared between all combinations to evaluate consistency associated with the modeling decisions.

## Results

### Comparison of combinations

There were differences between the average standard deviation of ranks across trials for all 12 combinations (Table 1), and most pair-wise comparisons between combinations were significantly different (not shown). Univariate combination #2 had the lowest variability in the univariate group (mean standard deviation = 36.6 positions), while multivariate combination #1 had the lowest variability in the multivariate group (mean standard deviation = 50.6 positions). These were chosen as the most promising combinations for further analyses, as described in the following section. Univariate combination #2 counted a repetition as significant at the 0.10 level without considering the direction of the effect. Multivariate combination #1 counted the number of variables that were significant at the 0.10 level when the effect was in the expected direction (Table 1).

**Fig. 2:**
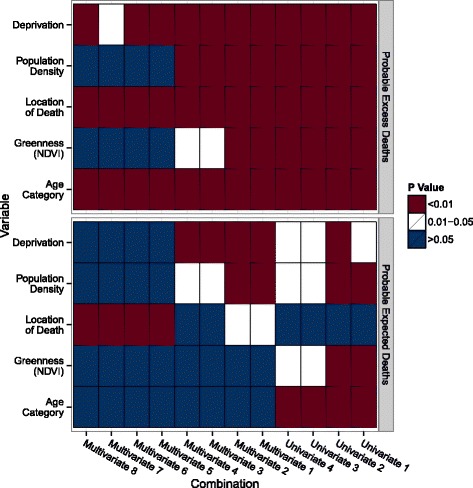
Heatmap comparing probable excess deaths, probable expected deaths, and controls for the five variables. Statistical analysis was performed comparing probable excess deaths (top) and probable expected deaths (bottom) from each combination with the pool of controls across the five variables. A *t*-test was used for greenness (NDVI), the Wilcoxon rank-sum test was used for the deprivation and population density variables, and chi-squared tests were used for the location of death and age category variables. Tests were repeated for all combinations. Color represents the significance level

The statistical differences between the probable excess deaths and the pool of controls were assessed for each of the five variables (Fig. 2). The probable excess deaths identified by the univariate combinations were significantly different from the pool of controls with respect to all variables. Those identified by the multivariate combinations #1–4 (in which the number of significant variables was tallied) were also significantly different from the controls for all variables, with the exception of greenness for combinations #3–4. Those identified by multivariate combinations #5–8 (in which the presence of at least one significant variable was tallied) did not differ from controls with respect to greenness or population density. Further, those identified by combination #7 (in which significance was tallied at the 0.05 level) did not differ from controls with respect to deprivation.

Statistical differences between the probable expected deaths and the pool of controls were also assessed for each of the five variables (Fig. 2), under the assumption that the differences would be small. The probable expected deaths identified by the univariate combinations were significantly different from the pool of controls with respect to all variables except location of death, though many of the p-values were in the 0.01 to 0.05 range. Those identified by the multivariate combinations #1–4 (in which the number of significant variables was tallied) were not different from the controls for the greenness and age category variables, and combinations #3–4 were not different for the location of death variable. Finally, those identified by multivariate combinations #5–8 (in which the presence of at least one significant variable was tallied) were only different from the controls for the location of death variable. Overall, it appears that differences were driven by one or two variables for each group of combinations.

**Fig. 3:**
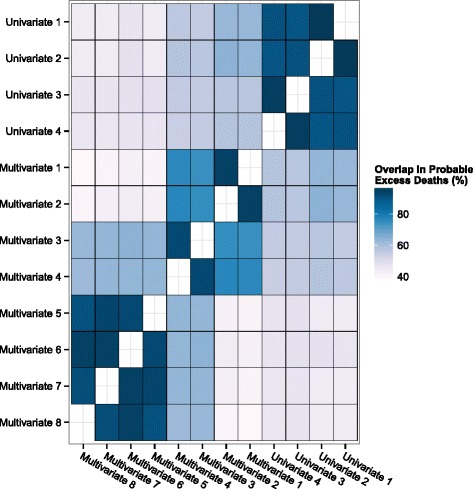
Percentage of overlap of identified probable excess deaths between combinations. Probable excess deaths were compared between each pair of combinations, and the percentage of overlap of individuals identified was plotted

The percentage of overlap in the probable excess deaths identified between each pair of combinations was evaluated (Fig. 3). Univariate combinations #1–4 showed strong internal consistency with overlap of >90% between all pairs. Multivariate combinations #1–4 showed similar internal consistency (mean overlap 81.9%), and so did multivariate combinations #5–8 (mean overlap 93.3%). The univariate combinations were more consistent with multivariate combinations #1–4 (mean overlap 58.0%), than with multivariate combinations #5–8 (mean overlap 45.6%). Multivariate combinations #3–4 were also consistent with multivariate combinations #5–8 (mean overlap 62.9%). Overall, there were 30 decedents (26.3% of the total 114) who were identified as probable excess deaths by all 12 of the modelling combinations.

### Further analyses on most promising combinations

Univariate combination #2 and multivariate combination #1 were selected for further analyses because they had the lowest variability in the standard deviations of rank (Table 1). Deaths from the pool of 411 cases were randomly selected into approximately 72% of the total five million repetitions (10,000 repetitions × 5 univariate models × 100 trials) for univariate combination #2, and one million repetitions (10,000 repetitions × 1 multivariate model × 100 trials) for multivariate combination #1. Deaths from the pool of 11,632 controls were selected an average of 10.2% of the time. In the univariate combination the mean ranks across 100 trials ranged from 3.9 to 410.3 (out of a possible 1.0 to 411.0) for the pool of cases, compared with 20.39 to 396.70 for the multivariate combination. The standard deviations of the ranks ranged from 1.1 to 54.3 positions for the univariate combination, and from 13.5 to 75.3 positions for the multivariate combination. The greatest consistency in rank was found in the highest and lowest ranked cases.

Distributions of the five variables were compared between the 114 probable excess deaths from the two selected approaches (Table 2). The deaths identified in the univariate approach were significantly younger, had a significantly lower residential greenness, and were significantly more deprived than those identified in the multivariate approach. No significant difference was found between approaches in the location of death or the population density. We also summarized the underlying causes of death for the probable excess deaths, according to categories of the ICD-10 (Table 2). The deaths identified by the univariate combination were shifted away from cardiovascular and respiratory causes compared with the multivariate combination, but shifted toward deaths from cancer and external causes starting with X in the ICD-10. The 17 probable excess deaths attributed to external causes in the univariate group included one highly-publicized death due to excessive natural heat and 16 deaths due to accidental poisonings by pharmaceutical or illicit agents and intentional self-harm. All but one of these deaths occurred at home or in the community.

**Table 2:** Summary statistics comparing the univariate #2 and multivariate #1 approaches, overlap cases, and the pool of controls

Variable	Description	Uni. #2 probable excess deaths	Multi. #1 probable excess deaths	*p*-value between uni. #2 and multi. #1 results	Overlap between uni. #2 and multi. #1	Overlap between all combinations	Pool of controls
N	Total number	114	114		72	30	11632
Age (%)	Age at death (%)						
	< 75 years	83.3	52.6	<0.001	79.2	96.7	37.4
	> = 75 years	16.7	47.4		20.8	3.3	62.6
Location of death (%)	Location of death (%)						
	In hospital	37.7	37.7	0.288	34.7	0	51.9
	Residential institution	17.5	27.2		19.4	0	30.9
	Home	36.0	28.9		37.5	80.0	14.5
	Other	8.8	6.1		8.3	20.0	2.7
Neighborhood deprivation quintile (%)	Neighborhood deprivation quintile (%)						
	1 (least deprived)	6.1	0	0.049	0	0	20.7
	2	4.4	0		0	0	20.7
	3	20.2	14.0		15.3	20.0	19.2
	4	21.9	33.3		26.4	13.3	19.1
	5 (most deprived)	47.4	52.6		58.3	66.7	20.2
Population density quintile (%)	Population density quintile (%)						
	0–181 persons/km^2^	1.7	0	0.829	0	0	20.1
	182–526 persons/km^2^	8.8	1.8		2.8	3.3	19.5
	527–681 persons/km^2^	10.5	18.4		8.3	16.7	19.9
	682–2356 persons/km^2^	28.9	30.7		33.3	20.0	19.8
	2357–2573 persons/km^2^	50.0	49.1		55.6	60.0	20.7
Greenness	Mean NDVI measurement	0.252	0.290	0.008	0.256	0.254	0.329
Selected underlying causes of death (%)	Underlying cause of death (%)						
	Cardiovascular (ICD-10 starting with I)	17.5	28.1	NA	22.2	36.7	27.1
	Respiratory (ICD-10 starting with J)	6.1	11.4		8.3	6.7	10.0
	Cancer (ICD-10 starting with C)	36.0	25.4		30.6	10.0	30.7
	External (ICD-10 starting with X)	14.9	8.8		13.9	26.7	3.1

Identified cases from the univariate and multivariate combinations were compared with respect to the five variables used in the analyses, as well as with respect to selected underlying causes of death. The overlap cases identified in both combinations (*N* = 72) and by all combi﻿nations (﻿*N *﻿= 30) were compared with the pool of controls

There were 72 probable excess deaths that were identified by both the univariate and multivariate combinations. Overall, 79% died at less than 75 years of age (compared with 37% among controls), 46% died in the community (17% among controls), 58% lived in the most deprived neighborhoods (20% among controls), 56% lived in the most densely populated neighborhoods (20% among controls), and there was lower vegetative greenness surrounding their residences. These differences were more pronounced when the probable excess deaths were restricted to the 30 identified by all 12 combinations (Table 2).

Finally, daily deaths during the extreme hot weather event ranged from 46 to 76, and all days were elevated above the summer 2009 mean of 42 (Fig. 1). Given that heat was assumed to be the underlying driver of increased mortality, we expected the probable excess deaths to be shifted towards the hotter days in the period. We assessed this by comparing the proportion of deaths on each day for: (1) all 411 deaths; (2) the 114 probable excess deaths identified by univariate combination #2 and by multivariate combination #1; (3) the 72 deaths identified by both combinations; and (4) the 30 deaths identified by all 12 combinations. The probable excess deaths were shifted towards the July 29–31 dates, and the shift was more pronounced among the 72 and 30 deaths that were identified by multiple modelling combinations (Table 3). Even so, all approaches had some probable excess deaths occurring on every day of the event.

**Table 3:** Summary of deaths by day of the 2009 hot weather event

Date, 2009	Daily maximum temperature at Vancouver International Airport (°C)	Total deaths (%)	Uni. #2 probable excess deaths (%)	Multi. #1 probable excess deaths (%)	Overlap between uni. #2 and multi. #1 (%)	Overlap between all combinations (%)
		*N* = 411	*N* = 114	*N* = 114	*N* = 72	*N* = 30
July 27	27.8	11.2	9.6	9.6	9.7	6.7
July 28	30.9	12.7	7.9	10.5	8.3	10.0
July 29	34.0	15.2	15.8	18.4	16.7	20.0
July 30	34.4	18.4	18.4	18.4	22.2	23.3
July 31	28.7	15.6	21.1	17.5	18.1	20.0
August 1	26.7	11.2	11.4	11.4	9.7	10.0
August 2	25.5	15.1	15.8	14.0	15.3	10.0

Columns show how the total deaths during this period compare with the most probably excess deaths identified by univariate combination #2, multivariate combination #1, the overlap between these combinations, and the overlap between all 12 combinations. The underlying assumption is that the most probable excess deaths would occur on the hotter days

## Discussion

Rapid epidemiologic assessment of mortality during extreme hot weather events is challenging because the excess deaths are almost always indistinguishable from the expected deaths in administrative vital statistics databases. Most excess deaths do not meet the criteria for certification due to excess heat [15], and are therefore attributed to the same ICD codes as the expected deaths. However, statistical tools that facilitate rapid epidemiologic assessment with these readily-available data could help generate timely evidence to protect vulnerable populations and to improve public health outreach during future events. The BCCDC has developed the proposed methods to identify the most probable excess deaths during a 2009 hot weather event in greater Vancouver, Canada. The approach is predicated on the assumption that the expected deaths during a hot weather event are similar to deaths that occur during normal summer temperatures when compared with respect to variables known to be associated with hot weather mortality. The framework for the approach is a case-control analysis into which hot weather cases and typical weather controls were randomly sampled from larger pools. Over thousands of repetitions and 100 trials, probable excess deaths were identified as cases that were most consistently found in non-null models.

Several sensitivity analyses were conducted to explore the behavior of the framework across variations in the input parameters including model type, significance weighting, coefficient directionality, and significance level. The excess deaths identified and the standard deviations of their ranking across 100 trials were most sensitive to the model type, where univariate and multivariable combinations were tested. In comparison, the direction of the significant coefficients had little impact on the stability of the results, suggesting that the effects were in the expected direction in the majority of repetitions. Similarly, there was little difference between the 0.10 and 0.05 significance levels, but the combinations with the 0.10 level showed more internal consistency.

All of the univariate combinations were considerably more stable than the multivariate combinations when assessed by the variability of the rankings. However, neither approach was completely consistent with our *a priori* assumption that the probable expected deaths would be similar to the pool of controls while the probable excess deaths would not (Fig. 2). There was also a marked lack of stability in the results from the multivariate combinations that counted a repetition as significant if any one of the five variables had a significant coefficient. This suggests that weighting the ranks for the number of significant variables in each repetition of the multivariable models produces more internal stability. Given that the model type was the most important choice, it is possible that other modelling or machine learning approaches would produce more robust results.

Overall, the suite of analyses demonstrated that the specification of the modeling framework affects the set of cases that will be identified as probable excess deaths and any subsequent conclusions drawn from the methods. Furthermore, we have used the ecologic time-varying deprivation, greenness, and population density variables as if their respective 2006, 2009, and 2010 values were static across the region during the 2009–2012 period. As such, both cases and controls may have been misclassified, which could bias the results if the actual exposures were systematically different from the estimates. For example, the City of Vancouver has planted many new street trees in recent years, meaning that the 2009 greenness estimates for the control population may be low when compared with the actual exposures. This could result in some cases with more moderate greenness values not being included in the group of probable excess deaths.

Another potential source of bias is the selection of the case and control periods. We defined the extreme event as July 27 through August 2 to be consistent with previous work [17], but this period includes 2–3 days when mortality was within the normal range of variability (Fig. 1). The results would have been considerably different if we had limited the pool of cases to the dates with significantly elevated mortality. Likewise, the results might be different if we had restricted the pool of controls such that we were randomly sampling the same proportion from the case group (297/411) and the control group (1,188/1,644) for each repetition. Ultimately, however, it would be ideal if we could test and refine this approach in a context where other, more conventional epidemiologic methods have been used to identify excess deaths. For example, Price et al. identified 106 of the 304 deaths as being heat-related via chart review after the 2010 event in Montreal [25]. Alternatively, the methods could be optimized using simulated data, in which the excess and expected deaths have been clearly defined. Either way, a dataset that included known excess deaths would allow refinement of the methods proposed here.

We have used deaths in the summers following the 2009 event as the pool of controls to simulate a conventional case-control study. However, the proposed methods could be most useful in the days and weeks following an extreme event associated with an obvious increase in mortality. In such cases the pool of controls would be drawn from a period before the event, as was done for our prior case-only analyses with these data [17]. Indeed, the ideal public health response could comprise the following steps: (1) rapid case-only analysis to ascertain variables most strongly associated with death during the extreme event; (2) application of the proposed methods to identify the most probable excess deaths; (3) review of charts/death certificates to confirm the role of extreme hot weather; and (4) targeted case-control study using administrative data or other methods, depending on the resources. However, the proposed approach is unlikely to be informative for short events with more subtle impacts, though it may be even more informative as the magnitude and/or duration of the exposure increases and its public health impacts are even further elevated above baseline.

The 30 cases identified by all 12 combinations were classified as the most probable excess deaths. These individuals were significantly younger, more likely to die in the community, and they lived in more densely populated, more deprived, and less green neighborhoods. Combined with the increase in external causes of death, which included deaths such as poisoning and self-harm, these results suggest that a marginalized population may be at highest risk in greater Vancouver. This is consistent with recent findings from the 2010 event in Montreal, where mental health illness was an important risk factor for death during the hot weather [25]. We cannot reliably evaluate the role of specific comorbidities or pharmaceuticals without access to death certificates or linkage between the vital statistics and other sources of administrative health data, but these results provide sufficient evidence to help target public health interventions during future episodes of extreme hot weather in the region.

## Conclusion

We have proposed and applied a method to probabilistically separate excess deaths from expected deaths during an extreme hot weather event. Many such events result in excess mortality that is not easy to characterize from administrative data. Once validated on data with known separation between excess and expected deaths, this method could be used to rapidly understand which deaths were most likely excess during any hot weather event and, therefore, to identify who is at most risk during future events in the region. The method may also be applicable to other episodic environmental hazards resulting in obvious excess morbidity or mortality. Although this method cannot replace conventional epidemiologic approaches, it may help to support public health protection when funding and/or access to more detailed data (ie medical charts) are not rapidly available for more rigorous study.

## Abbreviations

BC: British Columbia
BCCDC: British Columbia Centre for Disease Control
GWPv4: Gridded population of the world version 4
ICD: International classification of diseases
NDVI: Normalized difference vegetation index
VANDIX: Vancouver deprivation index
YVR: Vancouver International Airport
